# Associations between Anticholinergic Burden and Skeletal Muscle Indices and Physical Function in Older Adults: A Scoping Review

**DOI:** 10.31662/jmaj.2026-0062

**Published:** 2026-06-12

**Authors:** Daisuke Komiya, Kohji Iwai, Hirofumi Takeya, Kohei Aritomi, Keisuke Hatase

**Affiliations:** 1 Division of Rehabilitation, Omuta City Hospital, Fukuoka, Japan; 2 Division of Physical Therapy, Faculty of Rehabilitation and Care, Seijoh University, Aichi, Japan; 3 Department of Pharmacy, Omuta City Hospital, Fukuoka, Japan

**Keywords:** anticholinergic burden, frailty, older adults, skeletal muscle, activities of daily living

## Abstract

**Background:**

Anticholinergic burden has been linked to adverse geriatric outcomes. However, quantitative evidence regarding its associations with skeletal muscle indices and functional outcomes remains limited, particularly for muscle quality. We conducted a scoping review to map the current evidence and identify research gaps.

**Methods:**

Following the Preferred Reporting Items for Systematic Reviews and Meta-Analyses extension for Scoping Reviews, we searched PubMed, Google Scholar, and CiNii from inception to January 23, 2026 for English or Japanese primary observational or interventional studies that quantitatively assessed anticholinergic burden using validated or previously published scales/classifications and at least one outcome related to skeletal muscle (mass, quality, or strength), physical function, or activities of daily living (ADL)/instrumental ADL (IADL). Eligible populations were older adults (aged ≥60 years, or studies with a mean/median age ≥60 years, or studies reporting a predefined older-adult subgroup). Case reports, nonhuman studies, reviews, and editorials were excluded. Two reviewers independently screened records and charted data using a standardized form.

**Results:**

Of 231 identified records, 18 studies met the inclusion criteria. Two studies assessed skeletal muscle mass using bioelectrical impedance analysis, reporting appendicular skeletal muscle mass and skeletal muscle mass index; no study directly assessed skeletal muscle quality. Most studies evaluated physical function and/or ADL/IADL. The majority reported that higher anticholinergic burden was associated with poorer physical function and/or greater dependence in ADL/IADL, although some findings were subgroup-specific (e.g., restricted to older strata) or null for certain measures (e.g., grip strength and some Timed Up and Go analyses). Measures of anticholinergic burden and outcome ascertainment varied substantially across studies.

**Conclusions:**

Quantitative assessment of anticholinergic burden may serve as an auxiliary indicator to prompt medication review in older adults; however, the available evidence is associative and subject to residual confounding (including confounding by indication and polypharmacy). In contrast, evidence directly linking anticholinergic burden to skeletal muscle indices – especially muscle quality – remains scarce. Studies incorporating direct skeletal muscle assessments are needed to better characterize these associations and explore potential mechanisms, including indirect pathways involving the central nervous system, mood, and activity.

## Introduction

Many medications – including antidepressants, anxiolytics, antihistamines, antiparkinsonian agents, antipsychotics, and gastrointestinal drugs – contain components with anticholinergic properties and are widely prescribed to older adults ^(1)^. These drugs have been associated with adverse outcomes such as cognitive impairment, falls, fractures, and delirium ^(2), (3), (4), (5), (6)^. To comprehensively assess the cumulative effects of these agents, several anticholinergic burden scales have been developed and applied in both clinical research and practice, including the anticholinergic drug scale (ADS) ^(7)^, Drug Burden Index (DBI) ^(8)^, and anticholinergic cognitive burden (ACB) scale ^(9)^. Recently, the Japanese anticholinergic risk scale (JARS) was developed to reflect prescribing patterns specific to older adults in Japan ^(10)^.

The impact of anticholinergic burden on physical function is considered indirect, involving multiple pathways. Reduced skeletal muscle mass and muscle quality are commonly associated with muscle weakness, which is a key risk factor for falls and fractures in older individuals ^(11), (12), (13), (14)^. Anticholinergic drugs may be linked to poorer physical performance through plausible indirect pathways, including cognitive symptoms, mood changes, and reduced physical activity. Furthermore, an increased anticholinergic burden has also been linked to progression toward long-term care dependency ^(15)^. These observations suggest that quantitative assessment of anticholinergic burden may help identify older adults with a higher likelihood of functional decline and/or frailty and may inform preventive and rehabilitative efforts, while acknowledging that causal pathways remain to be clarified.

Most existing studies have reported fragmented findings related to functional indicators such as grip strength, gait speed, Timed Up and Go (TUG) test results, and activities of daily living (ADLs) ^(16), (17)^. In contrast, quantitative evidence linking anticholinergic burden to quantitative and structural muscle parameters remains very limited ^(18), (19)^. In this scoping review, we aimed to systematically map the current literature on the quantitative relationship between anticholinergic burden and skeletal muscle parameters (mass, quality, and strength), physical function, and ADL in older adults, to map current evidence, identify gaps in knowledge, generate insights for clinical applications, and propose directions for future research.

## Materials and Methods

### Study design and reporting guidelines

We conducted a scoping review to comprehensively map the existing literature on the association between anticholinergic burden and skeletal muscle-related indicators, namely muscle mass, muscle quality, muscle strength, physical function, and ADL in older adults. The review protocol followed the methodological guidance provided by the Joanna Briggs Institute (JBI) Manual for Evidence Synthesis ^(20)^, and reporting adhered to the Preferred Reporting Items for Systematic Reviews and Meta-Analyses (PRISMA) extension for Scoping Reviews checklist ^(21)^. The study protocol was preregistered with the Open Science Framework (OSF; registration number: kq96n; https://osf.io/kq96n). The preregistered protocol specified that the final literature search would be conducted on July 10, 2025; however, to incorporate evidence closer to submission and to improve transparency and reproducibility, we updated the searches on January 23, 2026, and operationalized database-specific screening limits for Google Scholar (including an explicit yield-saturation definition). These amendments are described in the Methods section (see ‘Information sources and search strategy’) and the Appendix 1.

**Appendix 1.** Search Strategies by Database.

Database	Search Terms
PubMed	(“Anticholinergic Agents”[Mesh] OR “anticholinergic burden” OR “drug burden index” OR “ACB scale”) AND (“Muscle, Skeletal”[Mesh] OR “Muscle Strength”[Mesh] OR “Sarcopenia”[Mesh] OR “muscle mass” OR “muscle quality” OR “sarcopenia” OR “skeletal muscle”) AND (“Aged”[Mesh] OR “older adults” OR “elderly” OR “geriatric”)
Google Scholar (Japanese)	(抗コリン薬 OR 抗コリン作用 OR 抗コリン薬負荷) AND (筋質 OR 筋肉の質 OR 筋密度 OR 筋肉のCT値 OR 筋肉のHU値 OR 筋エコー OR 低輝度筋 OR 筋減弱 OR BIA OR CT) [(“anticholinergic drug” OR “anticholinergic effect” OR “anticholinergic burden”) AND (“muscle quality” OR “quality of muscle” OR “muscle density” OR “muscle CT value” OR “muscle HU value” OR “muscle echo” OR “low-density muscle” OR “myosteatosis” OR BIA OR CT)]
Google Scholar (English)	(“anticholinergic burden” OR “anticholinergic drugs” OR “drug burden index” OR “ACB scale”) AND (“muscle quality” OR “muscle density” OR “CT attenuation” OR “Hounsfield Unit” OR “muscle echo” OR “low-density muscle” OR “muscle degradation” OR “sarcopenia” OR “BIA” OR “CT”)
CiNii	(抗コリン薬 OR 抗コリン作用 OR 抗コリン薬負荷) AND (筋質 OR 筋肉の質 OR 筋密度 OR 筋肉のCT値 OR 筋肉のHU値 OR 筋エコー OR 低輝度筋 OR 筋減弱 OR BIA OR CT) [(“anticholinergic drug” OR “anticholinergic effect” OR “anticholinergic burden”) AND (“muscle quality” OR “quality of muscle” OR “muscle density” OR “muscle CT value” OR “muscle HU value” OR “muscle echo” OR “low-density muscle” OR “myosteatosis” OR BIA OR CT)]

Filters and coverage applied: PubMed—no free full-text filter was applied; Humans; Languages: English/Japanese; Coverage: from database inception to January 2026 (last searched on January 23, 2026). Google Scholar—two queries (English and Japanese), each run once on January 23, 2026; screening was limited to the top 300 results per query (top 200 plus an additional 100 until yield saturation, defined as no newly eligible studies in the additional 100 results). CiNii—Japanese or English terms were applied as appropriate; screening was limited to the top 200 results per search; Coverage: from database inception to January 2026 (last searched on January 23, 2026). To minimize personalization effects in Google Scholar, searches were performed while logged out using Google Chrome in incognito mode after clearing browsing and search history. The preregistered OSF protocol specified that the final literature search would be conducted on July 10, 2025; searches were updated on January 23, 2026, and the database-specific Google Scholar screening limit/yield-saturation definition was documented as an amendment and reflected in the PRISMA flow diagram counts. ACB: Anticholinergic Cognitive Burden; BIA: bioelectrical impedance analysis; CT: computed tomography; HU: Hounsfield unit; MeSH: Medical Subject Headings.

### Eligibility criteria

The eligibility criteria were determined using the Population, Concept, Context (PCC) framework ^(22)^ as follows:

(1) Population: Older adults, operationally defined as individuals aged ≥60 years, or studies with a mean/median age ≥60 years, regardless of residential setting (community-dwelling, hospitalized, or institutionalized), health status, or country. Studies with broader age ranges or lower minimum age thresholds were eligible only if they reported results for a predefined older-adult subgroup (e.g., ≥65 or ≥75 years); in such cases, we extracted data from the older-adult stratum.

(2) Concept: Quantitative assessment of anticholinergic burden using validated or previously published anticholinergic burden scales/classifications (e.g., ACB scale, ADS, anticholinergic risk scale, DBI, JARS, Korean anticholinergic burden scale [KABS], CRIDECO anticholinergic load scale [CALS], or anticholinergic burden classification), with evaluation of at least one of the following outcomes:

• Muscle mass or quality assessed using objective methods (e.g., bioelectrical impedance analysis [BIA], computed tomography [CT], or magnetic resonance imaging [MRI]).

• Muscle strength and/or physical function (e.g., grip strength, lower-limb strength, TUG, or Short Physical Performance Battery [SPPB]).

• ADL and/or instrumental ADL (IADL) (e.g., Barthel Index [BI], or Lawton IADL).

(3) Context: Studies conducted in any setting (clinical, community, or long-term care) were included. We included observational studies and interventional studies, when available, that used primary data.

Case reports, animal or in vitro studies, and non-original articles (e.g., narrative reviews and editorials) were excluded. Eligible sources of evidence were limited to full-text publications in English or Japanese. No restrictions were applied regarding the year of publication in order to capture all available evidence. Compared with the preregistered OSF protocol (Population aged ≥65 years and example scales such as ACB/DBI/JARS), we amended the operational definition of older adults and expanded the list of eligible anticholinergic burden instruments to better reflect heterogeneity in the literature while retaining geriatric relevance; subgroup-only extraction was prespecified to avoid including predominantly younger populations. These criteria were selected to ensure a transparent and reproducible scope for the review.

### Information sources and search strategy

We searched the PubMed, Google Scholar, and CiNii databases. The search strategy was based on the “Population” and “Concept” components of the PCC framework. Appendix 1 lists the specific search terms used. For PubMed, we used a combination of Medical Subject Headings and free-text terms; no full-text availability filter was applied. For Google Scholar, we ran two queries (one in English and one in Japanese), each executed once on January 23, 2026. To minimize personalization effects, searches were performed while logged out using Google Chrome in incognito mode after clearing browsing and search history. Given the limited reproducibility of ranking algorithms, we screened the top 200 results for each query and then screened an additional 100 results (total 300). Because no newly eligible studies were identified in the additional 100 results, we judged that yield saturation had been reached and stopped screening. For CiNii, Japanese or English terms were applied as appropriate, and we screened the top 200 results for each search. We also hand-searched the reference lists of eligible articles. The searches covered all available records from database inception to January 23, 2026. The preregistered OSF protocol specified a final search date of July 10, 2025; this was updated to January 23, 2026, to incorporate evidence closer to submission. In addition, while the OSF protocol did not prespecify a database-specific stopping rule for Google Scholar, we operationalized the Google Scholar screening limit (top 200 plus an additional 100 until yield saturation) as an amendment to improve transparency and reproducibility.

### Study selection

The search results were compiled using Microsoft Excel, and duplicates were removed. For Google Scholar and CiNii, screening was limited to the prespecified sets described under ‘Information sources and search strategy’. Two reviewers independently performed a two-stage screening:

• First stage (title/abstract screening): Independent screening of titles and abstracts, excluding records that did not meet the eligibility criteria.

• Second stage (full-text screening): The full texts of potentially eligible records were retrieved and assessed for final inclusion.

**Figure 1.**
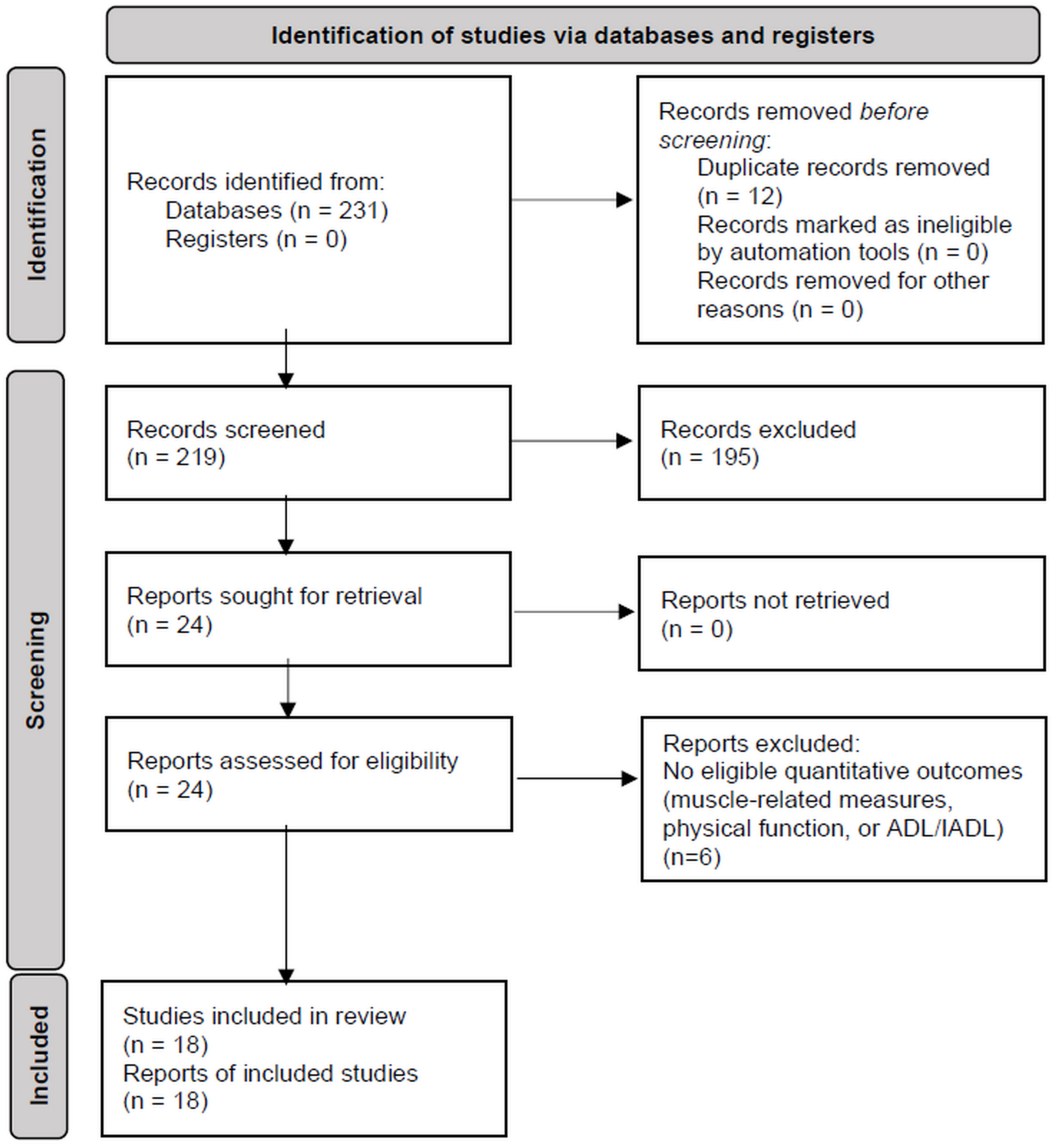
PRISMA 2020 flow diagram of the study selection process (searches last run on January 23, 2026). For Google Scholar, screening was limited to the top 300 records per query (top 200 plus an additional 100 until yield saturation) for one English and one Japanese query; for CiNii, screening was limited to the top 200 records per query. PRISMA: Preferred Reporting Items for Systematic Reviews and Meta-Analyses.

Discrepancies were resolved through discussion or adjudication by a third reviewer. The screening process is illustrated in the PRISMA 2020 flow diagram (Figure 1).

### Data extraction and synthesis

The two reviewers independently extracted data using a standardized Microsoft Excel sheet. Extracted data were cross-checked, and discrepancies were resolved by consensus. The extracted items included:

• Study characteristics: author, year, country, study design, sample size, and population details.

• Anticholinergic burden assessment: the scale used and the assessment method.

• Outcome evaluation: measures of muscle mass and/or quality, muscle strength, physical function, or ADL/IADL.

• Main findings: direction of association; whether findings were statistically significant after adjustment, including subgroup-limited or partial/limited significance, when applicable; key covariates used for adjustment; study limitations; and conflicts of interest.

The extracted data were summarized descriptively and organized by outcome domains, including muscle measures, physical function, and ADL/IADL. Tables were used to present study characteristics and outcomes. Consistent with the objectives of a scoping review and JBI guidance, we did not conduct a meta-analysis or a formal risk-of-bias assessment.

### Ethical considerations

This scoping review was based solely on publicly available literature and did not involve human participants or individual-level data. Therefore, institutional ethics approval and informed consent were not required.

## Results

### Source selection overview

Figure 1 presents the PRISMA 2020 flow diagram for study selection. A total of 231 records were identified, and 12 duplicates were removed. The remaining 219 records were screened by title and abstract, and 195 were excluded. The full texts of 24 records were assessed for eligibility. Of these, six were excluded because they did not report quantitative assessments of skeletal muscle indices (mass/quality/strength), physical function, or ADL/IADL. Accordingly, 18 studies were included in the final review.

### Source characteristics

The characteristics of the 18 studies are summarized in Table 1. These studies were published between 2009 and 2025 in the USA, Australia, Finland, the Netherlands, the UK, Ireland, Italy, France, Turkey, Malaysia, Japan, and South Korea ^(16), (17), (18), (19), (23), (24), (25), (26), (27), (28), (29), (30), (31), (32), (33), (34), (35), (36)^. Study designs included cross-sectional (n = 8), case–control (n = 1), retrospective cohort (n = 3), prospective/longitudinal cohort (n = 5), and mixed (cross-sectional + longitudinal) (n = 1). The sample size ranged from 115 to 42,132 (median, 649), reflecting both community-based and clinical cohorts.

**Table 1.** Characteristics of Included Studies: Design, Setting, Population, Anticholinergic Burden Measures, and Assessment of Skeletal Muscle Mass/Quality.

First author (year, country/region)	Design/setting	Population (n)	Anticholinergic measure	Muscle mass (method)	Muscle quality
Hilmer (2009, USA: Pittsburgh & Memphis)^16^	Longitudinal cohort (Health ABC)	2,172 (for SPPB longitudinal analysis)	DBI	No	No
Gnjidic (2009, Australia: Sydney)^23^	Cross-sectional (community cohort; older men)	1,705	DBI	No	No
Gnjidic (2012, Australia: Sydney retirement villages)^24^	Cross-sectional (baseline)	115	DBI (Beers also reported)	No	No
Gnjidic (2012, Finland: Kuopio)^25^	Cross-sectional (GeMS baseline)	700	DBI	No	No
Zia (2016, Malaysia: Kuala Lumpur)^26^	Case–control (recurrent/injurious falls vs controls)	428	ACB	No	No
O’Connell (2019, Ireland)^17^	Cross-sectional (older adults with intellectual disabilities)	677 total (functional tests subsets vary)	DBI, DBA, DBS	No	No
Yrjana (2020, UK: England)^27^	Prospective cohort (EPIC-Norfolk; cross-sectional & longitudinal analyses)	7,496	ACB	No	No
D’Alia (2020, Italy: multicenter)^28^	Prospective multicenter observational cohort (CRIME secondary analysis)	620	ACB	No	No
Attoh-Mensah (2020, France: Caen)^29^	Cross-sectional (community fallers ≥55 y)	177	ADS	No	No
Wouters (2020, Netherlands: LASA)^30^	20-year longitudinal cohort	2,627 (baseline)	DBI	No	No
Kose (2022, Japan: rehab ward)^31^	Retrospective cohort (post-stroke, convalescent rehab)	542	ARS	No	No
Bag Soytas (2022, Turkey: Istanbul)^18^	Cross-sectional (single-center; geriatric outpatient clinic)	256	ACB	Yes — BIA (Tanita TBF-300); SMMI (kg/BMI), sex-specific cut-offs	No
Erken (2023, Turkey)^32^	Cross-sectional (CKD outpatients vs controls)	321	ABC; Chew; DBI	No	No
Huh (2024, Korea: nationwide NHIS-Senior)^33^	Retrospective nationwide cohort	42,132	KABS	No	No
Tanaka (2025, Japan: Kashiwa)^19^	Longitudinal community cohort	1,549	Japanese Anticholinergic Drug Risk Scale	Yes — BIA (appendicular skeletal muscle mass)	No
Sürmeli (2025, Turkey: Ankara)^34^	Cross-sectional (community-dwelling ≥60 y)	521	ACB	No (probable sarcopenia via SARC-F + low grip only)	No
Muglia (2025, Italy)^35^	Cross-sectional & longitudinal (acute-care inpatients)	4,005	CALS; ACB	No	No
Komiya (2025, Japan: single acute-care hospital)^36^	Retrospective single-center cohort (hip-fracture surgery)	273	JARS (total ACB)	No	No

Key evidence gaps: No study quantitatively assessed skeletal muscle quality (e.g., CT attenuation, MRI fat fraction, ultrasound echo intensity). Two studies – Tanaka (2025) and Soytas (2022) – assessed skeletal muscle mass using BIA (ASMM and SMMI [kg/BMI]), respectively. Notes: One included study enrolled community fallers aged ≥55 years; it was retained because it reported age-stratified results for a predefined older subgroup (≥75 years), from which the key findings were extracted. ACB: Anticholinergic Cognitive Burden (scale); ABC: anticholinergic burden classification; ADS: Anticholinergic Drug Scale; ARS: Anticholinergic Risk Scale; ASMM: appendicular skeletal muscle mass; BIA: bioelectrical impedance analysis; CALS: CRIDECO Anticholinergic Load Scale; DBA: anticholinergic subscale of the Drug Burden Index; DBI: Drug Burden Index; DBS: sedative subscale of the Drug Burden Index; JARS: Japanese Anticholinergic Risk Scale; KABS: Korean Anticholinergic Burden Scale; SMMI: skeletal muscle mass index.

### Assessment of anticholinergic burden

Anticholinergic measures included DBI (n = 7), with the anticholinergic component (anticholinergic subscale of the Drug Burden Index and the sedative component [sedative subscale of the Drug Burden Index]) reported in one study, ACB (n = 6), JARS (n = 2), KABS (n = 1), anticholinergic risk scale (n = 1), ADS (n = 1), anticholinergic burden classification (n = 1), and CALS (n = 1) ^(35)^. Assessment sources varied and included self-report or interview with medication review, prescription/electronic medical record data in clinical cohorts, and claims databases (a nationwide cohort), with most studies applying quantitative scoring. However, the extent to which the dose was incorporated varied by scale.

### Outcomes: muscle and functional indicators

Two studies assessed skeletal muscle mass using BIA: one reported appendicular skeletal muscle mass (ASMM), and the other used a skeletal muscle mass index (SMMI). No study directly assessed skeletal muscle quality (e.g., CT- or MRI-derived muscle attenuation/fat infiltration). Physical function outcomes included grip strength (n = 11), usual gait speed or walking tests (n = 9), TUG (n = 5), SPPB (n = 2), and chair-stand tests (n = 4). Grip strength was evaluated as a primary outcome in 11 studies; one additional study used grip strength only for stratified analyses. In hospital-based cohorts, clinical functional outcomes such as the Functional Oral Intake Scale (FOIS) (n = 1), swallowing status using Section K3 of the interRAI Minimum Data Set for Acute Care (n = 1), and Functional Ambulation Categories (FAC) (n = 1) were also reported. ADL/IADL outcomes (e.g., BI, basic ADL (BADL), Lawton IADL, and functional independence [FI]) were assessed in seven studies.

### Associations between anticholinergic burden and outcomes

• Longitudinal evidence: Among the included studies with longitudinal or prognostic designs, higher anticholinergic burden was generally associated with worse subsequent outcomes (e.g., incident disability, decline in function, or other adverse events) when multivariable adjustment was performed. The strength of this evidence varied with the extent of confounding control; studies adjusting only for basic demographics (age/sex) were interpreted more cautiously than those additionally accounting for comorbidity burden, cognitive status, depressive symptoms, physical activity, and/or polypharmacy. Subgroup-only associations were reported in some cohorts (e.g., effects observed only in older strata [≥75 years], only among women, or only among individuals with low grip strength).

• Cross-sectional evidence: Cross-sectional and case–control analyses also tended to show directionally consistent associations between higher anticholinergic burden and poorer concurrent physical function and/or greater ADL/IADL dependence; however, temporality cannot be established, and residual confounding is more difficult to exclude. As with longitudinal studies, interpretability depended on the adjustment set, ranging from models controlling primarily for demographics to those additionally incorporating key geriatric confounders (e.g., comorbidities, cognition, mood, activity level, and medication burden).

• Subgroup-specific and null findings: Outcome-dependent null or limited findings were reported in several studies. For example, some analyses found no association for grip strength and/or TUG despite associations for other endpoints. In one study, there was no association with BIA-derived SMMI or gait speed despite significant associations with sarcopenia prevalence and BADL dependence. One study did not report a clear association after adjustment ^(32)^. ADL/IADL outcomes (e.g., BI, BADL, Lawton IADL, and FI) were assessed in seven studies, and reported associations—when present—were typically derived from multivariable-adjusted models. In addition to conventional performance tests such as SPPB, gait speed/walking tests, chair-stand tests, and TUG, hospital-based cohorts also evaluated clinical functional outcomes such as FOIS and FAC (each n = 1).

### Adjustments for confounding factors and study limitations

Table 2 summarizes the adjusted associations, key covariates, and concise findings for each study. The interpretability of reported associations varied with the extent of confounding control. Most analyses accounted for core confounders such as age, sex, adiposity (body mass index), comorbidity burden (Charlson Comorbidity Index or Functional Comorbidity Index), cognition (Mini-Mental State Examination or Cognitive Performance Scale), mood (Geriatric Depression Scale score or depression diagnosis), socioeconomic or lifestyle factors (education, smoking, alcohol use, and physical activity), nutritional or inflammatory status (Geriatric Nutritional Risk Index and C-reactive protein), kidney function (estimated glomerular filtration rate), fall history, and overall medication load/polypharmacy. However, the adjustment sets were not uniform across studies; some models included only basic demographics, whereas others additionally incorporated geriatric syndromes and medication burden, which affects the strength of inference. Sensitivity analyses were reported in three studies (alternative cut-offs or exposure definitions), and effect modification was examined in three studies by age, such as ≥75 years, sex, or low grip-strength subgroups. Across these analyses, the direction of reported associations was generally consistent after adjustment, although outcome-specific null findings and residual confounding remained possible. Common limitations included limited temporality in cross-sectional designs and exposure ascertainment, heterogeneity in anticholinergic burden scales, and variability in outcome measurement across settings.

**Table 2.** Physical Function and ADL/IADL Outcomes, Adjusted Associations with Anticholinergic Burden, and Key Findings.

First author (year, country/region)	Physical function outcomes	ADL/IADL outcomes	Association and direction	Significant after adjustment	Key covariates adjusted (examples)	Key finding
Hilmer (2009, USA)^16^	SPPB, usual gait speed, grip strength	—	Yes (negative)	Yes	Age, sex, race/site, education, comorbidity index, baseline function, cognition, mood, sleep, hospitalizations	Higher DBI at baseline and over follow-up predicted lower SPPB, slower gait, and lower grip at ~5 years.
Gnjidic (2009, Australia)^23^	Chair stands, 6 m gait speed, narrow-path gait, balance sway, grip	IADL scale	Yes (negative)	Yes	Age, education, BMI, falls, medical conditions, cognition, depression	Higher DBI is associated with slower gait, worse balance, lower grip, and more IADL difficulty; sedative load often showed stronger effects than anticholinergic load.
Gnjidic (2012, Australia)^24^	SPPB, grip	—	SPPB: Yes (negative); Grip: No	SPPB: Yes; Grip: No	Age, sex, education, comorbidity (FCI), MMSE, GDS, daytime sleepiness	DBI related to lower SPPB; no independent association with grip; Beers criteria did not predict performance.
Gnjidic (2012, Finland)^25^	10 m gait speed, 5× chair stand, TUG, grip	Lawton IADL, Barthel Index	Yes (negative) except Grip (No)	Yes (except grip)	Age, sex, education, FCI, MMSE, self-rated health, etc.	DBI>0 linked to slower gait, worse chair stands/TUG, and lower IADL/BI; grip strength showed no clear association.
Zia (2016, Malaysia)^26^	TUG, Functional Reach, grip	—	Falls: Yes (positive risk); Function: mixed (attenuated)	Falls: Yes; Function: mixed	Age, sex, comorbidity count; models including TUG/FR/grip	ACB is independently associated with recurrent/injurious falls; functional measures may mediate the relationship.
O’Connell (2019, Ireland)^17^	Grip, TUG	Barthel Index	Barthel: Yes (negative with DBS); Grip/TUG: No	Barthel: Yes; Grip/TUG: No	Age, ID level, falls, FCI, non-DBI meds; sex (subset)	Sedative burden (DBS) is associated with greater ADL dependence; no independent association with grip or TUG.
Yrjana (2020, UK)^27^	Usual gait speed, timed chair-stand speed, tandem balance, grip	—	Yes (negative)	Yes (sex-specific nuances)	Age, diseases, SES/education, smoking, alcohol, BMI, activity	Higher ACB predicted poorer mobility and balance longitudinally; effects were generally stronger in women.
D’Alia (2020, Italy)^28^	Grip (for stratification)	BADL (baseline covariate)	Mortality ↑ with high ACB only in the low-grip subgroup	Yes (in low-grip strata)	Age, sex, meds, CVD risks/diagnoses, cognition, mood, BADL, etc.	ACB≥2 predicted 1-year mortality among patients with low grip; the interaction ACB×grip was significant.
Attoh-Mensah (2020, France)^29^	TUG, grip; executive function (TMT)	—	≥75y: Yes (negative for TUG/performance)	Yes (age-stratified); TUG attenuated with TMT in the model	Age, education, BMI, falls risk, comorbidities, grip	In ≥75y, daily anticholinergic use is associated with impaired TUG and executive function; motor effect may be partly mediated by cognition.
Wouters (2020, Netherlands)^30^	Walking, Cardigan test, Chair stands, Balance	Functional Independence (self-report)	Partial: Yes (negative) for chair stands, upper-limb (Cardigan), FI; walking/balance NS	Partial (see left)	Sex, age, education, living status, BMI, depression, comorbidities, non-DBI meds	Long-term DBI exposure linked to poorer lower/upper limb performance and lower functional independence.
Kose (2022, Japan)^31^	Swallowing (FOIS)	FIM (baseline only)	Yes (negative; ΔARS↑→FOIS↓)	Yes	Age, sex, BMI, CRP, GNRI, stroke subtype, baseline FIM-T, med count	Increase in ARS during rehab is independently associated with worse FOIS at discharge.
Bag Soytas (2022, Turkey)^18^	Sarcopenia (BIA-based SMMI [kg/BMI] with sex-specific cut-offs), grip strength, 6-m gait speed	BADL, IADL	Yes (harmful)	Partial/limited	Age, BADL (and limited CGA-related covariates; adjustment for main outcomes was limited)	Higher ACB (≥1) was associated with higher sarcopenia prevalence and greater BADL dependency; grip strength showed a negative association (attenuated in multivariable analyses), whereas SMMI and 6-m gait speed were not significant. Cross-sectional design and exposure duration were not assessed.
Erken (2023, Turkey)^32^	4 m gait speed	—	No clear association reported	—	Age, eGFR, med count, nutrition, CCI, frailty	ABC is higher in CKD and related to frailty; a direct adjusted association with gait speed is not shown.
Huh (2024, Korea)^33^	TUG (≥10 s abnormal)	—	Yes (negative: higher KABS → TUG abnormal)	Yes	Sex, BMI, income, lifestyle, mood/cognition, ADL; +HTN/DM/DLP for mortality models	Higher KABS is associated with abnormal TUG; both high KABS and chronic polypharmacy predicted all-cause mortality.
Tanaka (2025, Japan)^19^	Frailty components: sarcopenia (grip, gait)	—	Yes (positive risk for incident frailty/sarcopenia)	Yes	Age, sex, education, income, BMI, living status, cognition, depression, activity, diet, drinking, chronic diseases, polypharmacy, PIMs	J-Anticholinergic score ≥3 doubled the risk of incident frailty and sarcopenia in community-dwelling older adults.
Sürmeli (2025, Turkey)^34^	Grip, gait speed	Katz ADL, Lawton IADL	Yes (negative)	Yes	Age, Charlson Comorbidity Index (examples)	Higher ACB associated with low strength/slow gait and greater ADL/IADL dependence; polypharmacy and frailty predicted high ACB.
Muglia (2025, Italy)^35^	Swallowing function (dysphagia status/change)	BADL	Yes (negative)	Yes	Age, sex, BADL impairment count, cognition (CPS), diagnoses, med count	Higher CALS/ACB associated with prevalent dysphagia, in-hospital worsening, and incident dysphagia; deprescribing at discharge linked to improvement.
Komiya (2025, Japan)^36^	Ambulation (FAC at discharge; ≤2 = assistance)	—	Yes (negative: higher JARS → assistance needed)	Yes	Age, albumin, fracture type, preinjury aid use, dementia meds; sensitivity adds BMI, timing/surgery, LOS, rehab dose	Higher Total ACB independently predicted assisted ambulation at discharge after hip-fracture surgery; exploratory age-stratified AUC was highest in 75–84 y.

Notes: Reported associations are from multivariable-adjusted models where available; significance and subgroup-specificity are indicated in the table. When an outcome shows no association or significance is limited to a subgroup (e.g., ≥75 years; low-grip subgroup), this is explicitly stated in the “Key finding (2–3 lines)” column. When the “Significant after adjustment” column indicates “Partial/limited,” this denotes that significance was outcome- or subgroup-specific and/or that multivariable adjustment for the primary outcomes was limited; details are provided in the “Key finding (2–3 lines)” column. ACB: Anticholinergic Cognitive Burden (scale); ADL: activities of daily living; ADS: Anticholinergic Drug Scale; ASMM: appendicular skeletal muscle mass; AUC: area under the curve; BADL: basic activities of daily living; BIA: bioelectrical impedance analysis; BI: Barthel Index; CALS: CRIDECO Anticholinergic Load Scale; CCI: Charlson Comorbidity Index; CHS: Cardiovascular Health Study frailty index; CPS: Cognitive Performance Scale; CRP: C-reactive protein; CVD: cardiovascular disease; DBA: anticholinergic subscale of the Drug Burden Index; DBI: Drug Burden Index; DBS: sedative subscale of the Drug Burden Index; DM: diabetes mellitus; DLP: dyslipidemia; eGFR: estimated glomerular filtration rate; FAC: Functional Ambulation Categories; FCI: Functional Comorbidity Index; FI: functional independence; FIM: Functional Independence Measure; FIM-T: total score of the Functional Independence Measure; FOIS: Functional Oral Intake Scale; FR: Functional Reach (test); GDS: Geriatric Depression Scale; HTN: hypertension; IADL: instrumental activities of daily living; JARS: Japanese Anticholinergic Risk Scale; KABS: Korean Anticholinergic Burden Scale; LOS: length of stay; MMSE: Mini-Mental State Examination; NS: not significant; PIMs: potentially inappropriate medications; SMMI: skeletal muscle mass index; SPPB: Short Physical Performance Battery; SES: socioeconomic status; TCSS: timed chair-stand speed; TMT: Trail Making Test; TUG: Timed Up and Go.

## Discussion

In this scoping review, we systematically mapped the literature on associations between anticholinergic burden and skeletal muscle mass, quality, and strength, physical function, and ADL in older adults. Two overarching observations emerged. First, the evidence directly linking anticholinergic burden to skeletal muscle structure is sparse: two studies assessed skeletal muscle mass using BIA, reporting ASMM or SMMI. Neither quantified muscle quality. Second, longitudinal evidence suggests that higher anticholinergic burden is associated with poorer subsequent functional outcomes and/or greater disability risk in some cohorts, whereas cross-sectional evidence is directionally consistent but limited by temporality. Across designs, the interpretability of reported associations varies with confounding control and remains vulnerable to residual confounding, including confounding by indication and polypharmacy; observed functional associations may also reflect indirect central nervous system (CNS)/mood/activity pathways rather than direct skeletal muscle toxicity. Together, these findings underscore a substantial evidence gap in studies that directly incorporate skeletal muscle structure—particularly muscle quality—alongside functional outcomes.

While associations with functional outcomes were frequently adjusted and directionally consistent, their biological interpretation warrants caution. The findings are more compatible with indirect pathways than with direct skeletal muscle toxicity. In particular, residual confounding—including confounding by indication and overall medication burden/polypharmacy—may partially explain the observed associations. Anticholinergic drugs likely influence CNS function, mood, and activity levels, thereby degrading performance on gait speed, TUG, SPPB, chair-stand, or grip strength tests and increasing dependence in ADL/IADL ^(16), (23)^. Related literature has suggested links with frailty ^(19)^ and progression to long-term care dependency ^(15)^, yet no included study demonstrated a causal chain from anticholinergic burden to structural muscle change. Accordingly, the observed associations should not be taken as proof of causality.

Several cross-cutting methodological issues further temper inference and external validity. First, database coverage was limited: due to constrained research funding, we were unable to access subscription databases such as Embase, CINAHL, Scopus, and Web of Science; therefore, some eligible studies may have been missed. Although we complemented database searches by screening Google Scholar/CiNii and hand-searching reference lists, incomplete coverage cannot be ruled out. Second, anticholinergic exposure was operationalized using heterogeneous instruments (ACB/ADS/Anticholinergic Risk Scale/CALS, DBI and its Drug Burden Index/Drug Burden Index subscales, KABS, JARS) with non-overlapping drug lists and variable handling of dose, formulation, and indication, complicating cross-study synthesis. Third, exposure timing was often cross-sectional: many studies relied on single medication lists, excluded as-needed medications or incompletely captured over-the-counter drugs, or did not quantify cumulative exposure, leaving temporality uncertain. Fourth, outcome ascertainment varied across settings (gait speed, TUG, SPPB, chair-stand, grip strength, BI/BADL, Lawton IADL, and FI), sometimes incorporating self-reporting and risking ceiling/floor effects. Fifth, selection and information biases may have affected generalizability: community cohorts often enrolled higher-functioning volunteers or single-sex/region samples, whereas hospital-based cohorts, such as hip-fracture or stroke-rehabilitation studies reporting FAC or FOIS, reflect acutely ill populations; claims or health-check cohorts (e.g., nationwide TUG) may under-ascertain exposures/outcomes and remain vulnerable to residual confounding despite extensive adjustment. Finally, evidence on skeletal muscle structure remains scarce: only two studies assessed muscle mass (both using BIA: ASMM or SMMI), and none quantified muscle quality (e.g., CT attenuation, MRI fat fraction, or ultrasound echo intensity), limiting mechanistic inferences regarding links between anticholinergic burden and muscle-related measures, particularly muscle quality.

Clinically, these results suggest that anticholinergic burden is a potentially relevant pharmacologic exposure associated with geriatric syndromes, aligning with the broader view that such syndromes – including physical frailty – are important outcomes considered in anticholinergic prescriptions ^(37)^. However, because the available evidence is associative and subject to residual confounding (including confounding by indication and polypharmacy), these findings should be interpreted as signals rather than proof of causality. Practical steps may include routine assessment of anticholinergic burden at admission (e.g., using JARS) as an auxiliary indicator to prompt multidisciplinary medication review when clinically appropriate, particularly in individuals with functional vulnerability. Observed functional associations may also reflect indirect CNS/mood/activity pathways and thus do not necessarily imply direct skeletal muscle toxicity. In older adults undergoing CT (e.g., after fractures, opportunistic evaluation of muscle mass and quality) may be feasible using existing scans ^(38)^. Future research should prioritize standardized, dose-weighted exposure definitions with repeated measures; align exposure windows with outcome assessments; incorporate direct, imaging-based measures of muscle quality; and employ prospective or interventional designs (e.g., deprescribing trials), with harmonized functional endpoints to clarify causality and improve generalizability.

## Conclusions

In this scoping review, higher anticholinergic burden in older adults was frequently associated with poorer physical function and greater dependence in ADL and IADL. However, studies directly incorporating structural muscle phenotyping remain limited. Only two studies quantified muscle mass, and none provided quantitative assessments of muscle quality, which constrains mechanistic inferences regarding links between anticholinergic burden and muscle-related measures, particularly muscle quality. Moreover, given heterogeneity in anticholinergic burden scales, uncertainty in exposure and outcome assessment, and the potential for residual confounding, causal interpretation should remain cautious. Clinically, anticholinergic burden is best positioned not as a stand-alone screening tool, but as an auxiliary indicator that can prompt medication review and multidisciplinary management. Future research should standardize exposure definitions (including dose weighting) with repeated measures, align exposure windows with outcome assessments, incorporate direct imaging-based evaluation of muscle quality, and employ prospective or interventional designs (e.g., deprescribing interventions). Evaluating incremental prognostic value beyond established functional measures such as gait speed and grip strength will further clarify clinical utility.

## Article Information

### Acknowledgments

The authors would like to express their sincere gratitude to all individuals who provided valuable input and support for this review. This study was conducted with institutional support from Omuta City Hospital and the Faculty of Rehabilitation, Seijoh University.

### Author Contributions

Conceptualization, project administration, methodology, investigation, writing – original draft: Daisuke Komiya. Writing – review & editing: Daisuke Komiya, Kohji Iwai, Hirofumi Takeya, Kohei Aritomi, and Keisuke Hatase. Supervision, Keisuke Hatase. All authors read and approved the final manuscript.

### Conflicts of Interest

None

### IRB Approval Code and Name of Institution

Ethical approval was not required for this study, as it involved an analysis of previously published literature. This review was conducted according to the research ethics policies of the authors’ institutions.

Patient consent was not required as this scoping review did not directly involve human participants and used only publicly available data.
